# De novo missense variants disrupting protein–protein interactions affect risk for autism through gene co-expression and protein networks in neuronal cell types

**DOI:** 10.1186/s13229-020-00386-7

**Published:** 2020-10-08

**Authors:** Siwei Chen, Jiebiao Wang, Ercument Cicek, Kathryn Roeder, Haiyuan Yu, Bernie Devlin

**Affiliations:** 1 Department of Computational Biology, Cornell University, Ithaca, NY 14853 USA; 2 Weill Institute for Cell and Molecular Biology, Cornell University, Ithaca, NY 14853 USA; 3 Department of Molecular Biology and Genetics, Cornell University, Ithaca, NY 14853 USA; 4 Department of Biostatistics, University of Pittsburgh School of Public Health, Pittsburgh, PA 15213 USA; 5 Department of Computer Engineering, Bilkent University, 06800 Ankara, Turkey; 6 Computational Biology Department, Carnegie Mellon University, Pittsburgh, PA 15213 USA; 7 Department of Statistics, Carnegie Mellon University, Pittsburgh, PA 15213 USA; 8 Department of Psychiatry, University of Pittsburgh School of Medicine, Pittsburgh, PA 15213 USA; 9 Present Address: Analytic and Translational Genetics Unit, Department of Medicine, Massachusetts General Hospital, Boston, MA 02114 USA

**Keywords:** Autism spectrum disorder, De novo missense variation, Protein–protein interaction, Cell-type-specific transcriptome

## Abstract

**Background:**

Whole-exome sequencing studies have been useful for identifying genes that, when mutated, affect risk for autism spectrum disorder (ASD). Nonetheless, the association signal primarily arises from de novo protein-truncating variants, as opposed to the more common missense variants. Despite their commonness in humans, determining which missense variants affect phenotypes and how remains a challenge. We investigate the functional relevance of de novo missense variants, specifically whether they are likely to disrupt protein interactions, and nominate novel genes in risk for ASD through integrated genomic, transcriptomic, and proteomic analyses.

**Methods:**

Utilizing our previous interactome perturbation predictor, we identify a set of missense variants that are likely disruptive to protein–protein interactions. For genes encoding the disrupted interactions, we evaluate their expression patterns across developing brains and within specific cell types, using both bulk and inferred cell-type-specific brain transcriptomes. Connecting all disrupted pairs of proteins, we construct an “ASD disrupted network.” Finally, we integrate protein interactions and cell-type-specific co-expression networks together with published association data to implicate novel genes in ASD risk in a cell-type-specific manner.

**Results:**

Extending earlier work, we show that de novo missense variants that disrupt protein interactions are enriched in individuals with ASD, often affecting hub proteins and disrupting hub interactions. Genes encoding disrupted complementary interactors tend to be risk genes, and an interaction network built from these proteins is enriched for ASD proteins. Consistent with other studies, genes identified by disrupted protein interactions are expressed early in development and in excitatory and inhibitory neuronal lineages. Using inferred gene co-expression for three neuronal cell types—excitatory, inhibitory, and neural progenitor—we implicate several hundred genes in risk (FDR $\le \hspace{0.17em}$0.05), ~ 60% novel, with characteristics of genuine ASD genes. Across cell types, these genes affect neuronal morphogenesis and neuronal communication, while neural progenitor cells show strong enrichment for development of the limbic system.

**Limitations:**

Some analyses use the imperfect guilt-by-association principle; results are statistical, not functional.

**Conclusions:**

Disrupted protein interactions identify gene sets involved in risk for ASD. Their gene expression during brain development and within cell types highlights how they relate to ASD.

## Background

Whole-exome sequencing studies of subjects diagnosed with autism spectrum disorder (ASD), their unaffected siblings, and their parents demonstrate significantly elevated rates of recurrent de novo variants in certain genes in ASD subjects [1–4]. For some genes, recurrence across ASD subjects is far more than expected by chance and is evidence for association with ASD. The evidence, however, largely comes from protein-truncating variants (PTVs) as opposed to de novo missense (dnMis) variants. These results are intuitive; PTVs, on average, should be more damaging. Yet, dnMis variants are more common. Within the 6430 ASD cases recently sequenced by the Autism Sequencing Consortium [5] (ASC), the ASC analyzed 7131 de novo variants in protein-coding exons, of which 4503 were missense (63.1%) and 972 were PTV (13.6%). For the ASC study, only the most damaging class of dnMis variants—as judged by a composite score involving evolutionary conservation and likelihood an amino acid substitution is damaging [6]—shows strong signal for enrichment in ASD subjects. These missense variants are uncommon, 8.3% of all missense variants given a score.

Using an earlier version of the ASC data, with roughly half as many de novo variants, we previously documented that rare dnMis variants that disrupt protein–protein interaction (PPI) are also enriched in ASD subjects [7]. Furthermore, these variants tend to affect proteins with many interactions, so-called hub genes, and their interactors tend to be ASD genes [7]. Using the newly released and larger ASC dataset, we confirm these observations and take them in several new directions: (1) By defining a set of genes encoding these disrupted protein interactors in ASD subjects and another for their siblings, we evaluate their expression patterns in developing brain from fetal to early postnatal development and within general cell types of brain tissue. Relative to the set defined by siblings, these analyses show that genes encoding disrupted protein interactions in ASC subjects tend to be expressed at higher levels and earlier in development and have the highest level of expression in neuronal lineages of both excitatory and inhibitory neurons. (2) Because these variants tend to disrupt interactions involving hub proteins in ASC subjects, we build a disrupted protein network using their disrupted interactions to define PPI network edges and a complementary non-disrupted network. By contrast to the non-disrupted network, the disrupted network shows significant enrichment for ASD proteins and this tends to increase with a greater number of interactions per protein. The latter observation can be used to implicate additional genes and sub-networks in risk for ASD. (3) Based on these results, we integrate hub information, gene expression for three neuronal cell types, and genetic association information using DAWN [8, 9] (detecting association with networks) to implicate new genes in risk for ASD. DAWN identified 421, 413, and 281 significant genes (FDR $\le \hspace{0.17em}$0.05) as candidate ASD genes for excitatory, inhibitory, and neural progenitor cells, ~ 60% of them are novel, and all sets show hallmarks of bonafide ASD genes. These sets point to neuronal morphogenesis and neuronal communication as critical for ASD risk, while for the neural progenitor cells, its DAWN set also shows strong enrichment for development of the limbic system. And, (4) we investigate these DAWN gene sets for whether their expression occurs across excitatory, inhibitory, and neural progenitor cells and which are unique to a particular cell type. This analysis reveals that the shared genes are enriched for previously identified genes highlighted by the recent ASC study [5], whereas genes whose expression is unique to a cell type tend to be enriched in genes implicated in ASD by other types of data. They also tend to function in a wide variety of roles in neuronal communication.

## Methods

### Interaction disruption prediction

A comprehensive set of high-quality physical interactions compiled in HINT [10] from eight widely used interaction databases (including BioGRID [11], MINT [12], iRefWeb [13], DIP [14], IntAct [15], HPRD [16], MIPS [17], and the PDB [18]) provides a structurally resolved 3D human interactome network. We evaluated the impact of dnMis variants on protein interactions by intersecting 6542 dnMis variants uncovered in a recent WES study from the ASC [5] from ASD probands and their unaffected siblings with 64,399 human protein interactions obtained from HINT. In total, we found 3822 dnMis variants—2922 in ASD probands and 900 in unaffected siblings—are on proteins with at least one known interaction within the current human interactome dataset, affecting 2364 probands and 737 siblings.

We employed a two-tiered predictive model we developed previously [7] to assess the probability a dnMis variant disrupts a protein interaction. For each dnMis variant, the model evaluates (1) whether it is likely to be on protein interaction interfaces and (2) whether it tends to have damaging functional effects on the protein. For (1), we applied an ensemble machine-learning algorithm (Interactome INSIDER [19], comprising the first full-proteome map of human interaction interfaces) to predict interface residues. For each variant, on each of its interactions with an interaction-specific partner, we considered a variant to be an interaction interface residue for this specific interaction if it has a probability score $\ge$“High” in Interactome INSIDER prediction. Next for (2), we evaluated its deleteriousness using PolyPhen-2 [20] (PPH2). If a variant predicted as an interface residue also has a “probably damaging” PPH2 score, we considered this variant to disrupt the interaction. Using our interaction disruption predictive model, we identified 123 unique dnMis variants that disrupt a total of 524 variant–PPI pairs in ASD probands and 26 unique variants disrupting 94 PPI pairs in siblings. These predicted disruptions involve 526 unique genes in probands. Of note, compared to our previous study, we tightened the tier (1) criterion from an interface score ≥ “ Medium” (yielding 388 candidates) to ≥ “High” (yielding 149 candidates).

### Published ASD gene lists

We compiled a total of 511 “previously implicated ASD genes” from two resources: (1) 102 TADA genes identified in our recent WES analysis [5] that carry a significant excess of disruptive variants in ASD subjects (henceforth TADA genes) and (2) 510 SFARI genes curated in the SFARI database (20200730 release, https://gene.sfari.org/database/gene-scoring/). Specifically, we use three categories and defined therein as category S (syndromic), genes with mutations that are associated with a substantial degree of increased risk and linked to additional characteristics not required for an ASD diagnosis; category 1 (high confidence), genes that have been implicated in ASD, typically with at least three de novo likely gene-disrupting mutations; and category 2 (strong candidate), genes with two de novo likely gene-disrupting mutations or a gene uniquely implicated by a genome-wide association study. This combined list was used for enrichment analyses, unless we note that TADA genes were excluded.

### Gene expression datasets

We analyzed the gene expression data from the BrainSpan atlas of developing human brain [21]. The BrainSpan dataset contains 607 bulk RNA-seq samples collected from 26 brain regions of 41 human subjects. We focused on the fetal and infant samples with age between 8 post-conception weeks (pcw) and 1 year, which includes 351 samples collected from 24 subjects. The majority of these subjects (16 of 24) are before 22 pcw. In the deconvolution analysis, we used a fetal scRNA-seq dataset [22] that includes 4261 human brain cells as a reference.

### Cell-type deconvolution and cell-type-specific (CTS) gene expression estimation

For cell-type deconvolution, we used a single-cell RNA-seq dataset of developmental human brain [22] and aggregated cells into seven major types using SC3 [23]: neuronal progenitor cell (NPC), excitatory neuron (ExN), inhibitory neuron (InN), microglia, astrocyte (Astro), oligodendrocyte (Oligo), and endothelial (Endo) cells. We selected the top 50 marker genes for each cell type with SC3 and then constructed a signature matrix by averaging the expression of marker genes across cells of the same type. We then used Multi-measure INdividual Deconvolution (MIND) [24] to estimate subject-level CTS gene expression by deconvolving the BrainSpan data. As a first step, MIND estimated cell-type fractions using nonnegative least squares with the pre-generated signature matrix derived from single-cell RNA-seq [22]. MIND borrows information from expression data across multiple measures, i.e., multiple brain regions per subject, and relies on empirical Bayes estimation to estimate CTS expression for each subject. It employs a computationally efficient EM algorithm to deconvolve all genes in the genome.

### CTS detecting association with networks (DAWN) analysis

We conducted DAWN [9] analysis for each neuronal cell type to integrate information of interaction disruption, CTS expression, and TADA score for autism risk. The analysis consists of two components: partial neighborhood selection to build CTS co-expression network based on MIND estimated subject-level CTS expression and a hidden Markov random field model to combine the estimated network with TADA score and disrupted hub identity as a covariate. With an FDR threshold of 0.05, we claimed CTS-DAWN genes significantly associated with autism risk.

### Gene ontology (GO) enrichment analysis

We performed GO enrichment analyses for CTS-DAWN genes directly on the GO home Web site (https://geneontology.org/, powered by PATHER [25]). In each test of a particular cell type, we customized the reference gene list to have a 5:1 match on the corresponding number of CTS-DAWN genes, using the optmatch [26] R package.

### CTSu-TADA gene co-expression analysis

We analyzed the gene co-expression between CTSu genes and two classes of TADA genes—58 GER- and 24 NC-TADA genes (GER: gene expression regulation, NC: neuronal communication, as pre-defined in our recent study [5]). A pair of CTSu-TADA genes was considered co-expressed if their expression has an absolute Pearson correlation coefficient ≥ 0.8 in the corresponding cell type. For each CTSu gene, the ratio of co-expressed GER- and NC-TADA genes expected at random is 58:24. We categorized a particular CTSu gene to be GER/NC enriched if it has a higher/lower GER:NC co-expression ratio than 58:24. We evaluated the enrichment using an exact binomial test and corrected for multiple testing using Bonferroni. In the correction, we only considered CTSu genes that have at least five co-expressed TADA genes, because when the number of co-expressed TADA genes is too small, a significance test is not meaningful: For the null hypothesis $Binomial$($x$, 58/(58 + 24)), the one-sided *P* value for one co-expressed TADA gene to be GER and $x\hspace{0.17em}$− 1 TADA genes to be NC can reach ≤ 0.05 only when $x\hspace{0.17em}\ge \hspace{0.17em}$5. Finally, 59 ExN-, 68 InN-, and 1 NCP-CTSu genes were kept for Bonferroni correction, yielding the corrected *P* values at 2.8 $\times \hspace{0.17em}$10^–5^, 2.8 $\times \hspace{0.17em}$10^–5^, and 0.05, respectively.

## Results

### Disruption of protein interaction helps identify important de novo missense variants in ASD probands

**Fig. 1:**
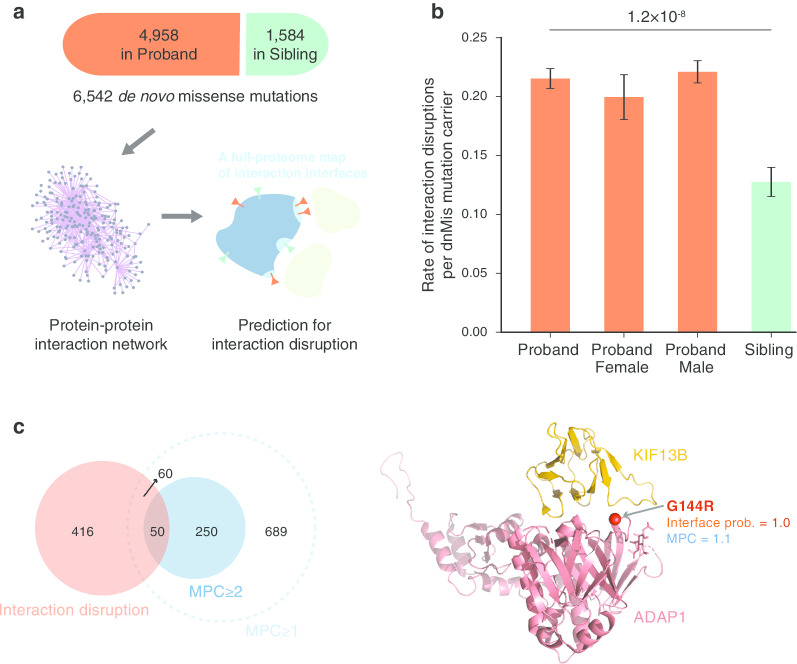
Disruption of protein interaction helps identify important de novo missense (dnMis) variants in ASD probands. **a** Data source of dnMis variants and a schematic diagram showing the proteome-wide mapping of dnMis variants onto protein–protein interaction interfaces for predicting interaction disruption. **b** Interaction disruptions are more common in ASD probands than in unaffected siblings. **c** Interaction disruption predictions rescue disruptive dnMis variants that were not recognized as damaging by MPC. Left: a Venn diagram showing the logical relations between genes affected by disruptive dnMis variants predicted by interaction disruption (red) and by MPC (Missense badness, PolyPhen-2, and Constraint; solid blue: MPC ≥ 2; dashed blue: MPC ≥ 1). Right: a co-crystal structure of ADAP1-KIF13B (PDB ID: 3MDB) displaying the interface location of an ASD proband dnMis variant ADAP1 G144R

We previously reported that disruption of protein interactions can contribute to ASD [7], and, by utilizing our first full-proteome interface mapping [19], we developed a computational approach to predict whether de novo missense (dnMis) variants disrupt protein–protein interaction (PPI) [7]. In brief, this approach predicts whether a particular residue is an interface residue using Interactome INSIDER. Then, if a dnMis variant falls in a predicted interface residue and is scored as “probably damaging” by PolyPhen-2, that variant was scored as interaction disrupting. Here, to study the functional relevance of dnMis variants and their disrupted interactions in ASD etiology, we applied our predictive approach to 6542 dnMis variants reported by a recent whole-exome sequencing (WES) study from the ASC [5] (Fig. 1a). We identified 123 unique dnMis variants predicted to disrupt the interaction of the encoded protein and at least one interacting partner in ASD probands and 26 unique variants in siblings (Additional file 2: Table 1).

To evaluate the impact of interaction disruption on ASD risk, we measured the differential rate of disrupted PPI by dnMis variants among ASD probands versus unaffected siblings. Of the 2364 probands who carry dnMis variants in genes encoding proteins known to interact—the interactome network—524 interactions were likely disrupted (0.22), as compared to 94 carried by 737 siblings (0.13). The rate of disruptions per subject was 1.74-fold higher in probands (*P* = 1.2 $\times \hspace{0.17em}$10^–8^ by a one-tailed *Z*-test, Fig. 1b) and recapitulated our previous finding that dnMis variants create risk for ASD, in part, by disrupting PPI. Rates of disrupted PPI per variant were more similar in ASD probands (524/123 = 4.3) and siblings (94/26 = 3.6), 1.18-fold, when compared to rates of disruptive variants (123/2922 = 0.042 and 26/900 = 0.029, yielding 1.46 fold). Thus, predictions of which dnMis disrupt interactions could serve as another effective approach for identifying damaging dnMis variants carried by ASD probands.

In their work, the Autism Sequencing Consortium [5] (ASC) used a composite metric called MPC [6] (combining Missense badness, PolyPhen-2, and Constraint) to separate damaging from benign dnMis variants. Specifically, for the ASC analyses, only missense variants with MPC ≥ 1 were considered sufficiently damaging to be entered into their TADA analyses. Thus, we investigated whether our prediction of disruptions of the PPI provided novel information beyond that provided by MPC. The two assessments were likely to be somewhat independent because, out of 123 genes in which dnMis variants were predicted to disrupt PPI, only 58.5% (72/123) had MPC ≥ 1. Considering interaction binding sites for these 123 and their unique interaction partners, there were 526 sites and 20.9% of them had MPC ≥ 1 (110/526), with a substantial fraction in the rarer and more highly deleteriousness category (MPC ≥ 2, Fig. 1c). Next, we fitted a logit model to evaluate the added impact of prediction of disrupted PPI beyond MPC status. For an outcome, we chose whether or not a gene was designated as involved in ASD risk, as determined by its presence in the list of SFARI genes curated for that purpose [27]. We fitted two variables to this outcome, whether a dnMis variant falling in a gene had a high MPC score (MPC ≥ 1, yes/no) and whether the variant disrupted a PPI involving that gene (yes/no). Because the SFARI list tends to include ASC-identified ASD genes, among others, the predictive value of variants with MPC ≥ 1 was of little interest, only whether the PPI predictions added predictive value. They did, a dnMis variant that was predicted to disrupt a PPI was 2.07 times more likely to occur in a SFARI gene even after accounting for MPC score (*z* = 2.68, *P* = 7.5 $\times \hspace{0.17em}$10^–3^). We further wondered if the PPI partners of genes carrying disrupting dnMis variants were also more likely to be SFARI genes. They were, whether MPC was taken into account (odds ratio = 1.77, z = 2.62, *P* = 8.$9\hspace{0.17em}\times \hspace{0.17em}$10^–3^) or not (odds ratio = 2.78, *z* = 4.98, *P* = 6.2 $\times \hspace{0.17em}$10^–7^). We next performed this analysis solely using data from less constrained genes [28] (with a pLI (probability of being loss-of-function intolerant) score < 0.9). For this subset and after adjusting for MPC, dnMis variants that disrupt PPI were still more likely to involve genes on the SFARI list (odds ratio = 3.72, *z* = 2.81, *P* = 5.0 $\times \hspace{0.17em}$10^–3^; for the disruption partner, odds ratio = 2.78, *z* = 2.84, *P* = 4.5 $\times \hspace{0.17em}$10^–3^). Finally, we asked whether dnMis variants that disrupt interactions were relatively more common in probands than their unaffected siblings, after controlling for MPC: They were (odds ratio = 1.42, *z* = 2.96, *P* = 3.1 $\times \hspace{0.17em}$10^–3^.)

These results suggest that our interaction-disruption approach could rescue disruptive dnMis variants that were not recognized as damaging by MPC. For example, we predicted a proband dnMis variant ADAP1 p.G144R (with a low MPC score of 1.1) to be disruptive to its interaction with KIF13B, with a very high interface probability score of 1.0 based on co-crystal structure analysis of ADAP1-KIF13B (Fig. 1c). The ADAP1-KIF13B interaction is known to function in regulating neuronal polarity formation and axon specification [29, 30], defects of which have been linked to ASD etiology [31, 32]. It is possible that the ADAP1 p.G144R variant contributes to ASD in the proband by disrupting ADAP1-KIF13B interaction, although the genetic evidence is still insufficient regarding this pair of genes.

### Transcriptome analyses of disrupted interactions implicate specific times and cell types in ASD

We next sought to characterize the potential functional relevance of disrupting interactions in the context of human neurodevelopment across developmental stages and cell types. To do so, we examined expression patterns of the genes for which dnMis variants disrupted PPI and their interaction partners, dubbed “disrupted interaction genes,” versus genes for which dnMis variants did not interrupt PPI. Here, we leveraged BrainSpan [21], a brain transcriptome database that provides bulk-tissue RNA-seq data from postmortem human brains across 26 brain regions and 16 periods of development from fetal to early postnatal. We also generated cell-type-specific (CTS) transcriptomes for seven cell types by deconvolving these BrainSpan bulk-tissue data using the Multi-measure INdividual Deconvolution (MIND) [24] algorithm.

**Fig. 2:**
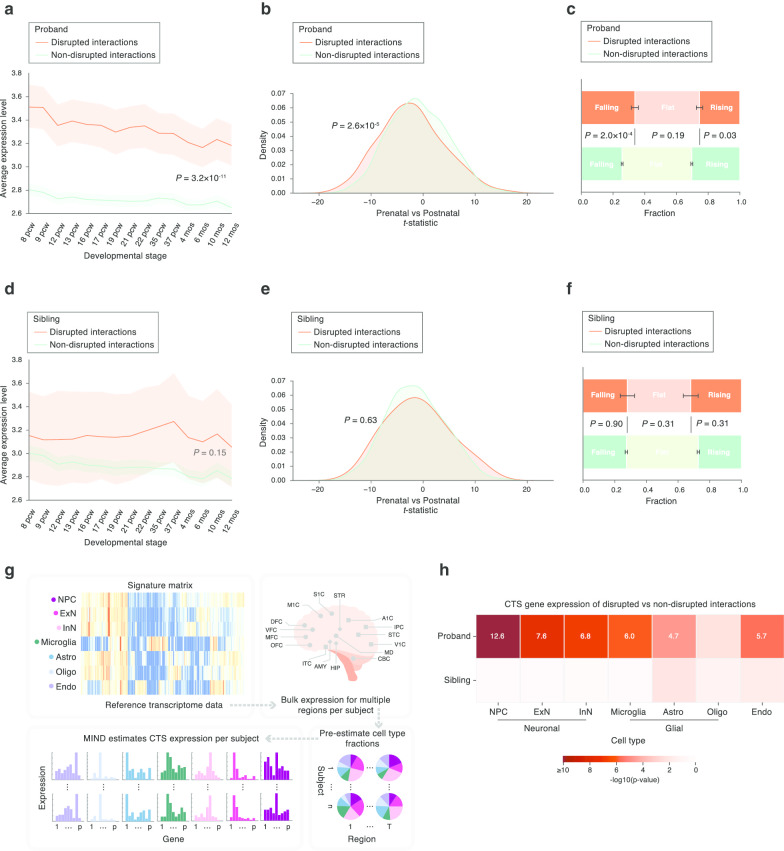
Transcriptome analyses of disrupted interactions implicate specific times and cell types in ASD. **a**–**c** Disrupted interaction genes identified in ASD probands **a** are highly expressed in brain, **b** exhibit prenatal expression bias, and **c** tend to have a falling expression trajectory. **d**–**f**, Disrupted interaction genes found in unaffected siblings do not present the above features. **g** MIND algorithm to estimate subject-level CTS expression from BrainSpan bulk RNA-seq data. **h** Disrupted interaction genes are most highly expressed in neuronal cell types. The heatmap shows the negative log10(*P* value) for comparing expression levels of disrupted versus non-disrupted interaction genes in seven cell types. Cell types that are significant after Bonferroni correction are noted with their negative log10(*P* value) written in white. *pcw* post-conception weeks, *mos* months, *CTS* cell-type-specific, *NPC* neuronal progenitor cell, *ExN* excitatory neuron, *InN* inhibitory neuron, *Astro* astrocyte, *Oligo* oligodendrocyte, *Endo* endothelial

Disrupted interaction genes were more highly expressed across brain development compared to non-disrupted interaction genes (*P* = 3.2 $\times \hspace{0.17em}$10^–11^ by a two-tailed *U*-test, Fig. 2a; see Additional file 1: Fig. 1 for parallel analyses removing known ASD genes). The contrast was more pronounced during earlier developmental stages, reflecting the reported prenatal expression bias of ASD risk genes [5, 33]. To further quantify this pattern, we applied a *t*-statistic that assessed the relative prenatal versus postnatal gene expression in developing brain (developed by the recent ASC study [5]). As expected, we observed a significant shift of disrupted interaction genes toward prenatal expression bias (smaller *t*-statistic than the non-disrupted interaction genes, *P* = 2.6 $\times \hspace{0.17em}$10^–5^ by a two-tailed *U*-test, Fig. 2b). Moreover, the BrainVar study [34] recently categorized genes based on their expression trajectories into “falling” (decreasing expression with age), “rising” (increasing expression with age), and “flat.” Consistent with the *t*-statistic results, disrupted interaction genes were more likely to have a falling expression trajectory (*P* = 2.0 $\times \hspace{0.17em}$10^–4^ by a two-tailed Fisher’s exact test, Fig. 2c). Of note, when we repeated all of these analyses for data from unaffected siblings, no significant test statistics were observed (Fig. 2d–f), reinforcing the functional significance of disrupted interaction genes we identified in ASD probands.

Another dimension we pursed to characterize interaction disruptions was at the cell-type level. This dimension can be particularly informative, given the vast cellular and functional diversity of neuronal and non-neuronal cell populations in brain tissue. Using MIND [24], we estimated subject-level CTS gene expression from the BrainSpan bulk data (Fig. 2g) for seven cell types: neuronal progenitor cell (NPC), excitatory neuron (ExN), inhibitory neuron (InN), microglia, astrocyte (Astro), oligodendrocyte (Oligo), and endothelial (Endo) cells. Comparing CTS expression of disrupted versus non-disrupted interaction genes, as identified by analysis of ASD probands, we found that disrupted interaction genes were expressed at a higher level across most cell types, in comparison with non-disrupted interaction genes. When the same analysis was applied to genes carrying dnMis variants found in unaffected siblings, this difference disappeared (Fig. 2h, two-tailed *U*-test, Bonferroni correction for seven tests). It was also evident that the neuronal cell types—NPC, ExN, and InN—showed more significant differences than any of the non-neuronal cell types, suggesting that the neuronal lineage could be more vulnerable than other cell types to interaction disruptions.

### A disrupted ASD network identifies novel ASD-associated proteins and protein interactions

**Fig. 3:**
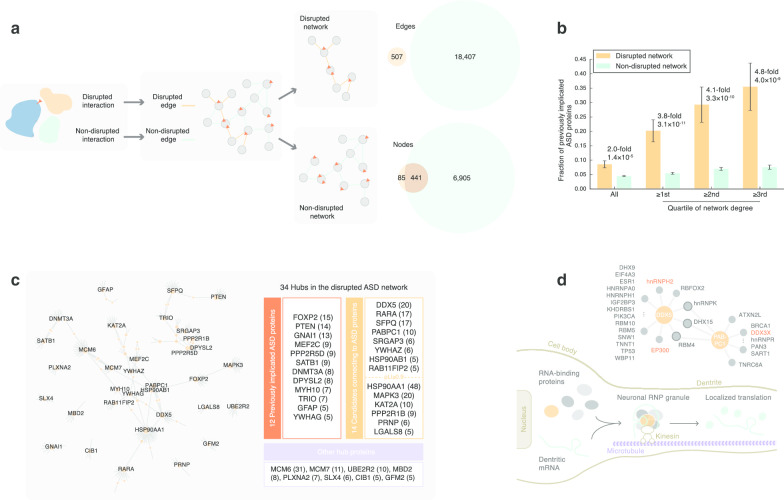
A disrupted ASD network identifies novel ASD-associated proteins and protein interactions. **a** Construction of disrupted and non-disrupted networks. **b** The disrupted network is enriched for previously implicated ASD proteins. Enrichment was assessed at four levels (from left to right): all proteins, proteins with a network degree higher than the first, the second, and the third quartile of corresponding network degree distributions. **c** Prioritizing disrupted hubs based on their degrees and connections to previously implicated ASD proteins. A network view of the 34 fourth-quartile hubs in the disrupted network is shown on the left (only hub genes are labeled). Numbers shown in the parenthesis indicate network degrees of hubs in the disrupted ASD network. **d** Prioritized proteins DDX5 and PABPC1 may function in the transport and localized translation of mRNAs encoding ASD proteins. Upper: a sub-network comprising proteins connected to DDX5 and PABPC1 in the disrupted network (red denotes previously implicated ASD protein). Lower: a schematic diagram illustrating the proposed roles of DDX5, PABPC1, and their interactors. RNP, ribonucleoprotein

A unique advantage of predicting which variants disrupt PPI resides in its ability to link different dnMis variants together by connecting these disrupted interactions. Toward this end, we created a “disrupted ASD network,” using the 507 disrupted interactions identified in ASD probands as PPI network edges (Fig. 3a, Additional file 3: Table 2). (We previously noted there were 524 interactions disrupted in probands, and here, we identify only unique protein pairs, reducing 524–507.) Similarly, a non-disrupted network was created using 18,407 non-disrupted interactions (Fig. 3a). Using these networks, we interrogated how 511 previously implicated ASD proteins were distributed (union of SFARI database [27], 20200730 release, and new genes recently implicated in risk for ASD [5]). Overall, there was a twofold enrichment of ASD proteins in the disrupted versus the non-disrupted network (44/526 versus 314/7346, *P* = 1.4 $\times \hspace{0.17em}$10^–5^ by a two-tailed *Z*-test). Interestingly, when we ranked the network proteins by their number of interactions or degree (low to high), we found an increasing enrichment at the higher degree (3.8-, 4.1-, 4.8-fold enrichment from the first to third quartile, respectively, Fig. 3b). This suggests that ASD proteins tend to act as hubs in our disrupted network. For this reason, we prioritized 34 hub proteins from the disrupted network, cutting at the highest degree quartile (≥ 5), as our candidate ASD proteins.

Of the 34 hub proteins, 12 fell in the set of 511 “ASD proteins” and the remaining 22 hub proteins did not. Of these 22 hub proteins, 14 were directedly connected to proteins falling in the 511 ASD protein list and eight of these 14 were encoded by genes that are extremely intolerant of loss-of-function variants (pLI [28] ≥ 0.9, Fig. 3c). DDX5 topped the list as having the highest network degree, with a dnMis variant DDX5 p.D255H disrupting 20 interactions. The majority of these disrupted interactors (17/20), as well as DDX5 itself, possessed RNA binding activity, and they were largely involved in mRNA splicing and transport (Fig. 3d). In particular, DDX5 and its interactor hnRNPA0 (heterogeneous nuclear ribonucleoprotein A0; gene *hnRNPA0*) are part of the FMRP-kinesin transport RNP granule [35], which travels along neuron dendrites and regulates the localized translation of mRNAs, some of which encode ASD proteins [36] (Fig. 3d). Additionally, besides hnRNPA0, three other hnRNPs are also among the DDX5 p.D255H disrupted interactors, including a previously implicated ASD protein hnRNPH2 [27]. Moreover, three interactors—RNA-binding proteins hnRNPK, DHX15, and RBM4—act as connectors linking DDX5 to another hub protein PABPC1 (p.K138E) in the disrupted network (Fig. 3d). Interestingly, PABPC1 is also a component of FMRP-associated RNP complex [37]. Previous experiments have suggested an indirect RNA-mediated interaction between DDX5 and PABPC1 [38], and in vivo experiments have further shown that knockout mice with DDX5 and PABPC1 disassociated from polyribosomes displayed ASD-relevant behaviors [39]. Collectively, together with published evidence, our disrupted ASD network nominates DDX5 and PABPC1 as novel ASD proteins.

### Integrating hub, CTS, and genetic information to implicate new genes in ASD risk

The ASC’s recent analyses of the same WES data have implicated 102 ASD genes that bear an excess of putatively damaging variants [5]. The evidence for genetic association of each gene was quantified by a TADA score, a Bayesian association testing metric that, in a recent instantiation, incorporated pLI score for protein-truncating variant (PTV) and MPC score for missense variant [5, 40]. Our as well as others’ transcriptome analyses [33, 41] have suggested that functional interpretation of ASD genes requires knowledge not only of the genetic changes on these genes but also of the neurodevelopmental context in which these changes function. Thus, toward enhancing the set of genes implicated in ASD and their interpretation, we integrated our new hub and CTS information with TADA into a unified framework detecting association with networks (DAWN) [8, 9] to implicate new genes for ASD in a CTS context.

**Fig. 4:**
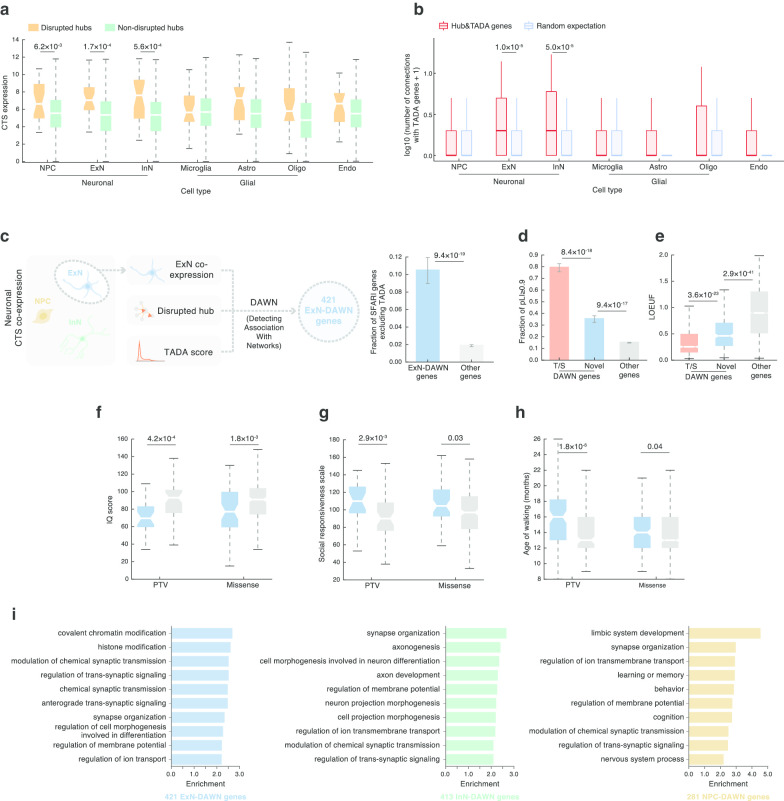
Integrating hub, CTS, and genetic information to implicate new genes in ASD risk. **a**, **b** Disrupted hubs are highly **a** expressed and **b** co-expressed with ASD genes in neuronal cell types. Cell types that are significant after Bonferroni correction are noted with their *P* values. **c** A unified framework DAWN integrating CTS co-expression, hub, and TADA to identify ASD genes in a CTS context. Three CTS-DAWN analyses were performed for three neuronal cell types. For presentation purposes, **c**–**h** present results of ExN-DAWN, and results of InN- and NPC-DAWN are provided in Additional file 1: Figs. 2 and 3, respectively. **d**, **e**, ExN-DAWN genes versus other genes evaluated on two loss-of-function tolerance metrics: pLI (**d**) and LOEUF (**e**). T/S stands for TADA/SFARI genes also identified by DAWN, Novel for DAWN genes not found in the T/S list, Other genes for expressed genes not found in the T/S or DAWN lists. **f**–**h**, ASD probands carrying a de novo protein-truncating or missense variant on ExN-DAWN genes exhibit severe ASD symptoms, with **f** reduced intelligence, **g** impaired social ability, and **h** delayed age of walking. **i** CTS-DAWN genes are enriched in neurodevelopmental processes. The top 10 enriched GO terms of each cell type are shown (FDR ≤ 0.05, ranked by fold-enrichment). *CTS* cell-type-specific, *ExN* excitatory neurons, *InN* inhibitory neurons, *NPC* neural progenitor cells, *TADA* transmission and de novo association, *DAWN* detecting association with networks, *pLI* probability of being loss-of-function intolerant, *LOEUF* loss-of-function observed/expected upper bound fraction, *PTV* protein-truncating variant

Our DAWN framework casts the ensemble data as a hidden Markov random field, in which the graph structure is determined by CTS gene co-expression, and it combines these interrelationships with node-specific observations, namely hub identity and TADA score, to detect correlated genes that reinforce ASD association signal. We chose the three neuronal cell types (NPC, ExN, and InN) as the relevant CTS context based on our previous finding that the neuronal lineage had the highest susceptibility to interaction disruptions (Fig. 2h); the ASC reached similar conclusions in their recent work [5]. We further confirmed and expanded on this by analyzing CTS expression and co-expression of hubs and TADA genes. Comparing CTS expression of disrupted versus non-disrupted hub genes revealed significantly higher expression of disrupted hubs in all three neuronal cell types, but in none of the others (NPC: *P* = 6.2 $\times \hspace{0.17em}$10^–3^, ExN: *P* = 1.7 $\times \hspace{0.17em}$10^–4^, InN: *P* = 5.6 $\times \hspace{0.17em}$10^–4^; Fig. 4a). In ExN and InN, moreover, disrupted hub and TADA genes were found more closely connected to each other via CTS co-expression (significantly denser than expected; ExN: *P* = 1.0 $\times \hspace{0.17em}$10^–5^, InN: *P* = 5.0 $\times \hspace{0.17em}$10^–5^; Fig. 4b). These results reinforced that neuronal cell types, especially ExN and InN, were likely to be the major cell types preferentially affected in ASD. In this regard, we performed three separate DAWN analyses for ExN, InN, and NPC, by fitting in the corresponding CTS co-expression network (Fig. 4c).

With an FDR ≤ 0.05, we identified 421, 413, and 281 significant genes as candidate CTS ASD genes for ExN, InN, and NPC, respectively (denoted as ExN/InN/NPC-DAWN or together as CTS-DAWN genes; Additional file 4: Table 3). Compared to other nonsignificant genes, and after removing the TADA genes [5], the remaining CTS-DAWN genes were significantly enriched for previously implicated SFARI ASD genes (Fig. 4c and Additional file 1: Fig. 2–3), lending support to the validity of our CTS-DAWN framework. Our framework implicated a substantial number of novel ASD genes. In each cell type, ~ 60% of CTS-DAWN genes were novel (not in previous TADA or SFARI gene list; Additional file 4: Table 3).

To validate the CTS-DAWN genes further and evaluate their functional significance, we performed a set of analyses for features known to be indicative of ASD genes, although *using only the novel CTS-DAWN genes*. First, we interrogated whether disruption to these genes was likely to have a severe impact on evolutionary fitness. Using the pLI [28] and loss-of-function observed/expected upper bound fraction (LOEUF) [42] metrics, we found that novel CTS-DAWN genes, on average, had a significantly higher pLI and correspondingly lower LOEUF scores than other genes (Fig. 4d, e and Additional file 1: Fig. 2–3). This reflected a strong selection against de novo variants on CTS-DAWN genes, such that a single disruptive variant could be enough to render a phenotypic effect (“haploinsufficient”). Next, we wondered if such variants had detectable effects on ASD-relevant phenotypes. To test this, we collected and compared several ASD diagnostic features between probands carrying variants in these genes versus probands carrying variants in genes not in DAWN or SFARI/TADA genes. We found that probands who carry a variant on novel DAWN genes, be it a PTV or a missense variant, exhibited reduced intelligence (low IQ score, Fig. 4f), impaired social ability (high social responsiveness scale, Fig. 4g), and delayed age of walking (Fig. 4h; Additional file 1: Fig. 2–3 and Additional file 5: Table 4). In sum, these analyses show that the CTS-DAWN genes share key features of SFARI/TADA ASD genes; namely, they are loss-of-function intolerant and they influence key ASD-associated phenotypes. It also suggests there is value of incorporating CTS co-expression information into DAWN analyses to discover novel ASD genes, which may be undetected by other methodologies using genetic association scores alone.

We next sought clues for the biological processes CTS-DAWN genes affect. Gene ontology (GO) enrichment analyses identified predominant signals from processes of neuronal morphogenesis (e.g., axonogenesis, neuron projection morphogenesis) and neuronal communication (synaptic transmission/signaling, synapse organization) over the three cell types (Fig. 4i, Additional file 6: Table 5). These processes are inter-related and have been implicated in ASD and associated syndromes [43–46]. NPC-DAWN genes showed a pronounced enrichment for “limbic system development” (Fig. 4i), which is intriguing because the limbic system is critical for emotion, behavior, memory, and learning [47], and abnormalities of the limbic system have been frequently associated with ASD [48], especially in the hippocampus and the amygdala. Moreover, it is interesting that this enrichment was only seen in NPC-DAWN genes, suggesting a very early developmental vulnerability for the limbic system.

### CTS co-expression drives the discovery of novel CTS-DAWN genes

**Fig. 5:**
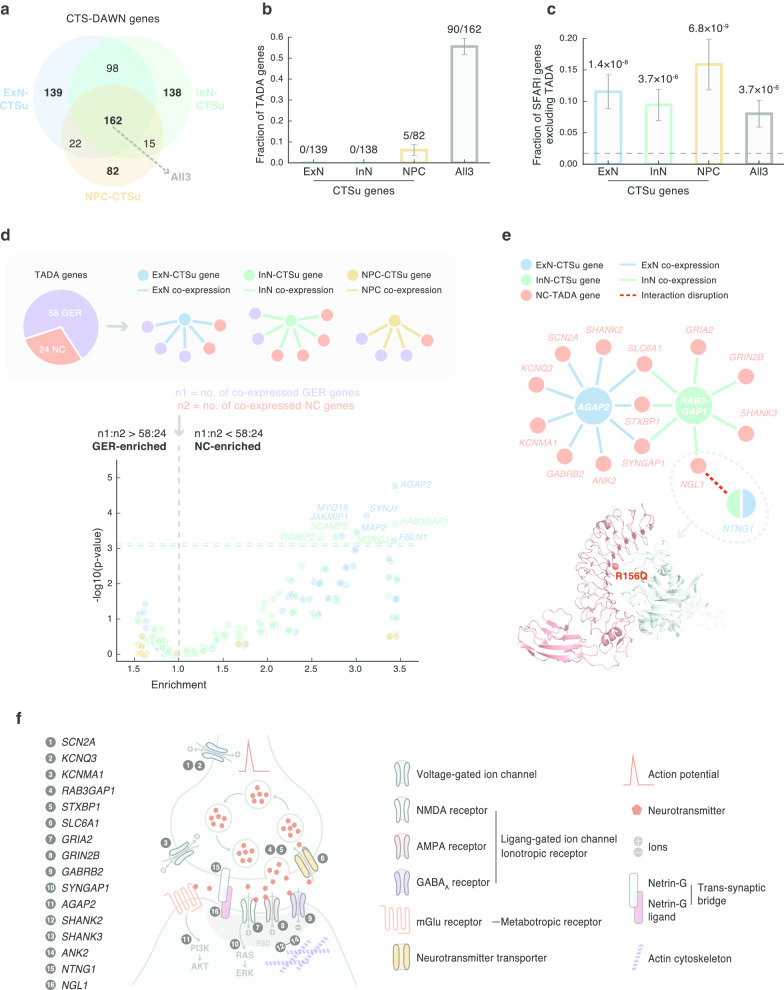
CTS co-expression drives the discovery of novel CTS-DAWN genes. **a** CTS-DAWN identifies genes that are unique to a particular cell type. **b** Overlaps between CTS-DAWN genes and TADA genes. **c** Overlaps between CTS-DAWN genes and SFARI genes. TADA genes were excluded from the analysis. Dashed line indicates the fraction of nonsignificant genes in DAWN analysis that overlap with SFARI genes. **d** CTSu-DAWN genes tend to be co-expressed with TADA genes involved in neuronal communication. Upper: A schematic diagram illustrating the co-expression analysis of CTSu-DAWN genes and TADA genes. Lower: A volcano plot showing the degree to which each CTSu-DAWN gene is GER enriched (left $x$-axis) or NC enriched (right $x$-axis), against corresponding statistical significance on $y$-axis (negative log10(*P* value)). Horizontal dashed lines indicate the threshold for significance after Bonferroni correction; names of genes that passed the correction are shown. **e** A condensed NC sub-network formed by inter-connected CTSu-DAWN genes and TADA genes. A co-crystal structure of NGL1-NTNG1 (PDB ID: 3ZYJ) displaying the interface location of an ASD proband dnMis variant NGL1 p.R156Q is shown below. **f** A schematic diagram showing the distribution and function of NC sub-network genes on neuron structures. The 16 NC sub-network genes function in a range of components of an NC circuit: (1) *SCN2A*, (2) *KCNQ3*, and (3) *KCNMA1* encode voltage-gated ion channels; (4) *RAB3GAP1* and (5) *STXBP1* assist neurotransmitter exocytosis, while (6) *SLC6A1* promotes restoration; (7) *GRIA2* and (8) *GRIN2B* encode glutamate receptors, and (9) *GABRB2* encodes a GABA_A_ receptor; in receptor-mediated pathways, (10) *SYNGAP1* suppresses NMDAR-RAS signaling and (11) *AGAP2* transmits signals from mGlu receptor to PI3K; in postsynaptic density (PSD), (12) *SHANK2*, (13) *SHANK3*, and (14) *ANK2* connect membrane receptors to the actin cytoskeleton; (15–16) the NTNG1-NGL1 interaction forms a trans-synaptic bridge. Abbreviations: CTS, cell-type-specific; DAWN, detecting association with networks; CTS-DAWN genes, genes identified by DAWN in specific cell types; ExN, excitatory neurons; InN, inhibitory neurons; NPC, neural progenitor cells; CTSu, cell-type-specific unique; ExN/InN/NPC-CTSu: genes identified uniquely in one cell type; All3, genes identified in all three cell types; TADA, transmission and de novo association; GER, gene expression regulation; NC, neuronal communication; NC-TADA, genes implicated by TADA and affect neuronal communication

To dissect how CTS information has contributed to CTS-DAWN discoveries, we compared the results across the three CTS-DAWN analyses. This produced four subsets of particular interest: three sets of genes that were unique to each of the three cell types (CTS-unique, abbreviated as “CTSu”) and one set of genes that were shared by all three cell types (“All3”; Fig. 5a). Comparing these four sets of genes with previous TADA genes, we found minimum overlap between the three sets of CTSu genes and TADA genes, whereas over half of the All3 genes overlapped with TADA genes (Fig. 5b). This sharp contrast can be attributed to and explained by the architecture of our CTS-DAWN framework. As the TADA score was an invariant input to all three CTS-DAWN analyses, genes with very high TADA scores were likely to be picked by all three. On the other hand, genes with relatively low TADA scores had to borrow strength from CTS gene co-expression. Technically, from the view of DAWN, a low-TADA-score gene will be promoted if it has a strong co-expression correlation with one or more high-TADA-score genes. Thus, we reason that the discovery of non-TADA CTSu genes was largely driven by their high co-expression with TADA (or high-TADA-score) genes. Interestingly, when we repeated the comparison using an independent set of ASD genes (SFARI excluding TADA), we found CTSu genes showed higher percentage representation than that of All3 genes (Fig. 5c). This, in part, follows from our earlier result, which shows DAWN genes tend to be over-represented in the SFARI gene list, even after excluding TADA genes (Fig. 4c).

To provide more biological meaning for new CTSu genes, we built on a recent ASC analysis [5], which classified TADA genes into two functional classes: 58 “gene expression regulation (GER)” genes and 24 “neuronal communication (NC)” genes. We next identified, for each CTSu gene, what specific TADA genes were co-expressed with it. We then assessed, for each CTSu gene, its tendency to function as a GER or NC by comparing the ratio of its numbers of co-expressed GER versus NC genes to the expected ratio (Fig. 5d). Our analyses identified similar proportions of GER- and NC-enriched genes (43% and 57%, respectively; Additional file 7: Table 6), although the NC axis yielded stronger statistical signals (Fig. 5d).

Along the NC-enriched axis, six ExN-CTSu (*AGAP2*, *SYNJ1*, *MAP2*, *MYO16*, *JAKMIP1*, and *FBLN1*) and four InN-CTSu (*RAB3GAP1*, *NDRG3*, *SCAMP2*, and *RIMBP2*) genes appeared significant after correction for multiple testing (Fig. 5d). Remarkably, the top genes *AGAP2* (in ExN) and *RAB3GAP1* (in InN) showed exclusive NC co-expression (9/9 and 7/7, respectively), and they were connected to each other through three common NC-TADA genes, thereby forming a local NC sub-network (Fig. 5e). The network was further condensed by an interaction-disruption event that linked one of the NC-TADA genes *NGL1* (with a dnMis variant p.R156Q) to an additional CTS-DAWN gene *NTNG1* (Fig. 5e). This clustering of different association signals from genetic association scores and their transcriptomic/interactomic interrelationships reinforced a convergence of CTS risk genes in NC. Mapping this network of genes onto neuronal structures, we found, intriguingly, they cover many essential components of an NC circuit (Fig. 5f). The genes affect action potential initiation and propagation (e.g., genes encoding voltage-gated ion channels: *SCNA2, KCNQ3, and KCNMA1*); neurotransmitter release/recycle at the pre-synapse (e.g., genes promoting neurotransmitter secretion [49, 50] and restoration [51] [*RAB3GAP1, STXBP1*, and *SLC6A1*]); neurotransmitter receptor binding at the post-synapse, from ionotropic receptors (e.g., glutamate receptors encoded by *GRIA2* [NMDAR] and *GRIN2B* [AMPAR] and GABA_A_ receptor encoded by *GABRB2*); ionotropic/metabotropic receptor-mediated intracellular signaling (e.g., *SYNGAP1* in NMDAR-RAS [52]; *AGAP2* in mGluR-PI3K [53]); and postsynaptic density (PSD) that connects membrane receptors to the actin cytoskeleton [4] (e.g., the Shank family [54]: *SHANK2/3*; the ankyrin family [55]: *ANK2*). Furthermore, the protein interaction between the presynaptic NTNG1 and the postsynaptic NGL1 forms a trans-synaptic bridge that mediates initial contact between early neuronal synapses [56, 57], which lays the foundation for NC. Collectively, we suggest that NC is likely to be a converging point for many of our CTS risk genes to function and that alteration of their gene expression and/or protein structure could have a neurodevelopmental and neuropsychiatric impact.

## Discussion

Our results show that de novo variants in ASD subjects alter nucleotides encoding protein-interaction interfaces more than expected by chance, often disrupting interfaces for multiple proteins, and the proteins involved in these disrupted interactions are more likely to be encoded by previously implicated ASD genes. Both the proteins encoded by the genes hit with dnMis variants and their complementary interactor proteins show this enrichment. These observations pave the way for a rich set of analyses involving gene sets identified by disrupted PPI, their gene expression during fetal to early postnatal development and within general cell types of brain tissue to determine how they relate to risk for ASD. PPI networks also hold key information in this regard, and guilt by association [58] in all these settings can implicate additional genes/proteins in risk for ASD.

This wide spectrum of analyses and results provides the following insights into ASD genetics and neurobiology: Genes encoding disrupted protein interactions in ASD subjects tend to be expressed earlier in development and in neuronal lineages of both excitatory and inhibitory neurons, in agreement with an earlier ASC study [5]. A PPI network built using dnMis variants disrupting hub protein interactions in ASD subjects shows significant enrichment for ASD proteins. By integrating hub information, gene expression for three neuronal cell types—excitatory, inhibitory, and neural progenitor cells—and genetic association information using DAWN [8, 9], we implicate three substantial sets of genes in risk for ASD (FDR ≤ 0.05), one for each cell type. Roughly, 60% of the genes in all three sets have not been implicated in ASD previously; yet, all sets and novel genes therein have characteristics of genuine ASD genes [1–3, 5]. Gene set analyses show all three sets are enriched for functions underlying neuronal morphogenesis and neuronal communication. Enrichment for these broad functions is a common finding for ASD genetics [1, 5, 43, 59]. Gene set analysis of DAWN genes from neural progenitor cells, however, also highlights strong enrichment for development of the limbic system.

While the genes identified by the DAWN analyses do show features of ASD-implicated genes, the evidence is not foolproof. Notably, the constraint scores for the novel DAWN genes fall between that of the DAWN genes also contained in the TADA/SFARI list and other genes not found in either source (Fig. 4d). If all genes involved in ASD risk were exactly of the nature of those found in the recent ASC manuscript [5], then a substantial fraction of the novel DAWN genes could be false positives. On the other hand, they could all affect risk, even though they are, on average, less constrained, because DAWN does not make the same assumptions about the genetic architecture of ASD as does TADA. TADA assumes a dominant model and works well for haploinsufficient risk genes. It cannot detect other kinds of ASD risk genes: those that are often lethal when mutated, are haplosufficient, or act in a sub-additive fashion, perhaps because of redundant mechanisms for development. In contrast, DAWN only assumes that ASD risk genes work together in a network, presumably for a common developmental purpose, and that a portion of those risk genes carry genetic association signal. In our analyses, that signal is generated by TADA-based association. Thus, DAWN targets a larger set of ASD risk genes than TADA.

## Limitations

Our analyses indicate that CTS gene co-expression and PPI networks can provide valuable insight into neurobiology relevant for ASD and they can implicate additional genes in risk for ASD. We recognize, however, that the guilt-by-association principle such analyses rely on is imperfect [60], as is the human interactome on which protein interactions and PPI networks are derived. The fact that implicated DAWN ASD genes have the hallmarks of validated ASD genes is strong evidence that many of the genes in these sets are real. The fact that they are enriched for neuronal functions previously implied in ASD is further evidence for their validity.

## Conclusions

Sample size for whole-exome studies of various diseases and disorders is constantly increasing. In most settings, however, de novo missense variants contribute only modestly to gene discovery and to the biology underlying the disorder [5]. Ideally, as sample size increases, so does our understanding of which missense variants seriously damage protein function and increase risk for disorders. Here, we show that dnMis variants that disrupt protein interactions are one dimension by which such variants affect risk for ASD. In our previous work on far smaller samples, we showed this is a general phenomenon across many disorders [7]. The importance of protein interactions and missense variation that disrupts them goes far beyond gene discovery, as we show here. Incorporating PPI and CTS co-expression networks promotes the identification and interpretation of risk genes under specific cell-type contexts. Disruptive missense variants shed light on genotype–phenotype relationships and gene sub-networks affecting risk. For ASD, we believe such sub-networks have the potential to identify key circuits involved in risk.

## Supplementary information

**Additional file 1.** This file contains supplementary table legends and figures.

**Additional file 2: Table 1.** Interaction-disruption predictions of dnMis variants (.xlsx). 524 dnMisvariant–PPI pairs identified in ASD probands and 94 pairs identified in unaffected siblings are listed. Foreach interaction disruption, we show the protein that harbors the dnMis variant and the amino acid changecaused by the variant, the interaction partner of the protein with dnMis variant, and the subject who carriesthe dnMis variant.

**Additional file 3: Table 2.** The ASD disrupted network (.xlsx). 526 nodes (proteins) and 507 edges(protein–protein interactions) composing the ASD disrupted network are listed. Nodes are ordered first bywhether the protein is a previously implicated ASD protein (in TADA/SFARI gene list, “1” for yes and “0”for no) and then by their degrees in the disrupted network.

**Additional file 4: Table 3.** Cell-type-specific (CTS) DAWN genes (.xlsx). 421, 413, and 281 significantCTS-DAWN genes identified in ExN, InN, and NPC are listed, respectively. In each cell type, CTS-DAWNgenes are ordered by their FDR values and are indicated whether they have been previously implicated inASD (in TADA/SFARI gene list, “1” for yes and “0” for no).

**Additional file 5: Table 4.** Diagnostic scores of ASD probands carrying CTS-DAWN gene variants(.xlsx). IQ score, social responsiveness scale, and age of walking of probands carrying a PTV or missensevariant on CTS-DAWN genes are listed.

**Additional file 6: Table 5.** Gene ontology (GO) enrichment results of CTS-DAWN genes (.xlsx). GOterms enriched for CTS-DAWN genes (significant after multiple testing correction) are listed.

**Additional file 7: Table 6.** CTSu-TADA gene co-expression in GER and NC (.xlsx). GER- and NC-enrichedCTSu genes together with the corresponding co-expressed GER/NC-TADA genes are listed.

## Data Availability

All results from analyses are presented in the supplementary tables.

## Abbreviations

ASC: Autism Sequencing Consortium
ASD: Autism spectrum disorder
Astro: Astrocyte
CTS: Cell-type-specific
CTSu: CTS-unique
DAWN: Detecting association with networks
dnMis: De novo missense
Endo: Endothelial
ExN: Excitatory neuron
GER: Gene expression regulation
GO: Gene ontology
InN: Inhibitory neuron
LOEUF: Loss-of-function observed/expected upper bound fraction
MIND: Multi-measure INdividual Deconvolution
MPC: Missense badness, PolyPhen-2, and Constraint
NC: Neuronal communication
NPC: Neuronal progenitor cell
Oligo: Oligodendrocyte
pLI: Probability of being loss-of-function intolerant
PPI: Protein–protein interaction
PSD: Postsynaptic density
PTV: Protein-truncating variant
TADA: Transmission and de novo association
TADA genes: Genes implicated in ASD risk by TADA analysis
WES: Whole-exome sequencing
