# Supplementary material for: De novo missense variants disrupting protein–protein interactions affect risk for autism through gene co-expression and protein networks in neuronal cell types
Source: Mol Autism. 2020 Oct 8;11:76. doi: 10.1186/s13229-020-00386-7 (PMC7545940; doi:10.1186/s13229-020-00386-7)
Supplement: Supplementary file 1 — Additional file 1. This file contains supplementary table legends and figures. [file 13229_2020_386_MOESM1_ESM.pdf]

Supplementary table legends

Supplementary Table 1: Interaction-disruption predictions of dnMis variants (.xlsx). 524 dnMis variant-PPI pairs identified in ASD probands and 94 pairs identified in unaffected siblings are listed. For each interaction disruption, we show the protein that harbors the dnMis variant and the amino acid change caused by the variant, the interaction partner of the protein with dnMis variant, and the subject who carriers the dnMis variant.

Supplementary Table 2: The ASD disrupted network (.xlsx). 526 nodes (proteins) and 507 edges (protein-protein interactions) composing the ASD disrupted network are listed. Nodes are ordered first by whether the protein is a previously implicated ASD protein (in TADA/SFARI gene list, "1" for yes and "0" for no) and then by their degrees in the disrupted network.

Supplementary Table 3: Cell-type-specific (CTS) DAWN genes (.xlsx). 421, 413, and 281 significant CTS-DAWN genes identified in ExN, InN, and NPC are listed, respectively. In each cell type, CTS-DAWN genes are ordered by their FDR values and are indicated whether they have been previously implicated in ASD (in TADA/SFARI gene list, "1" for yes and "0" for no).

Supplementary Table 4: Diagnostic scores of ASD probands carrying CTS-DAWN gene variants (.xlsx). IQ score, social responsiveness scale, and age of walking of probands carrying a PTV or missense variant on CTS-DAWN genes are listed.

Supplementary Table 5: Gene Ontology (GO) enrichment results of CTS-DAWN genes (.xlsx). GO terms enriched for CTS-DAWN genes (significant after multiple testing correction) are listed.

Supplementary Table 6: CTSu-TADA gene co-expression in GER and NC (.xlsx). GER- and NC-enriched CTSu genes together with the corresponding co-expressed GER/NC-TADA genes are listed.

Supplementary figures

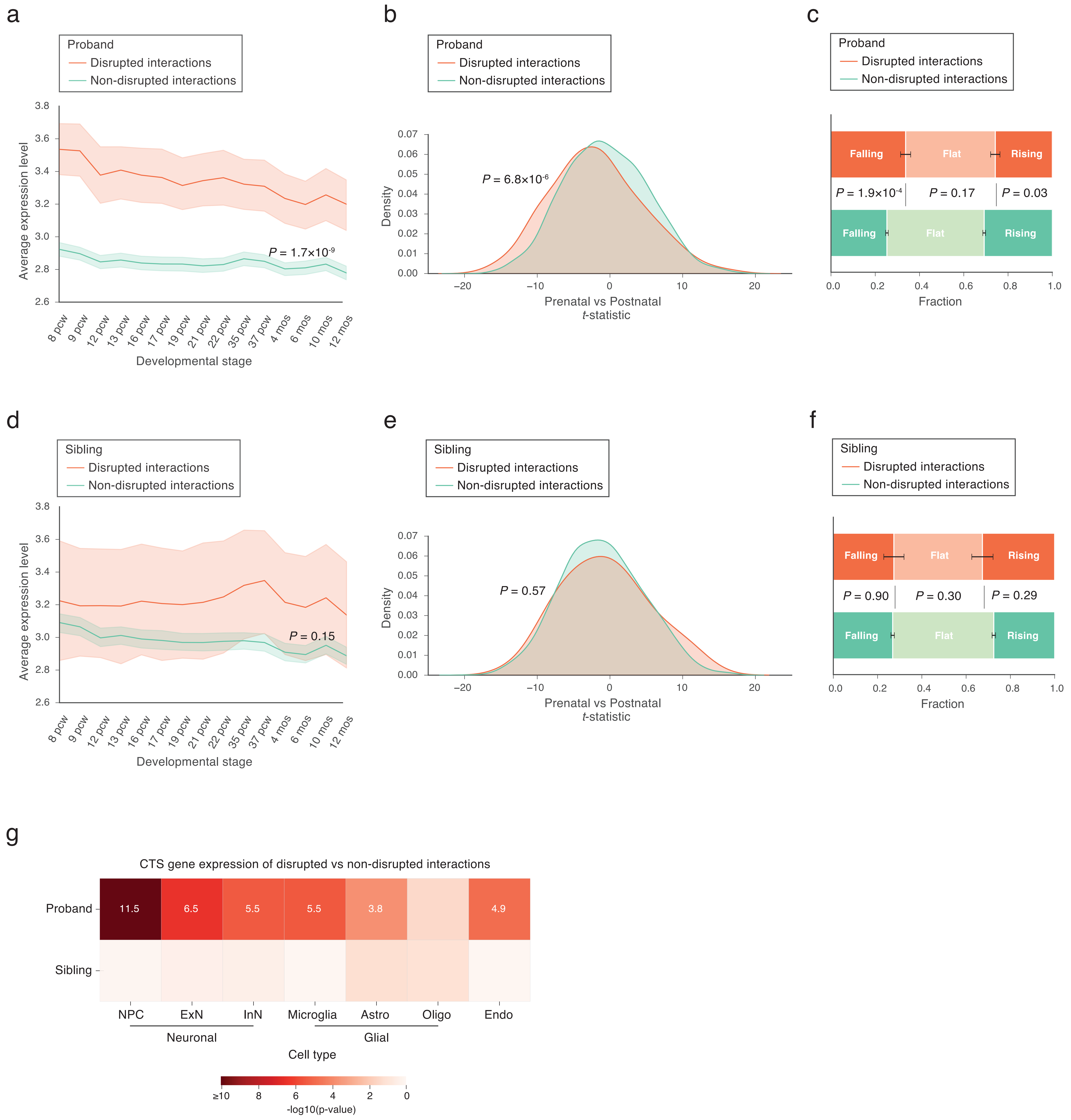

Supplementary Fig. 1: Transcriptome analyses of disrupted interactions with known ASD genes excluded. **a-c**, Disrupted-interaction genes identified in ASD probands (**a**) are highly expressed in brain, (**b**) exhibit prenatal expression bias, and (**c**) tend to have a falling expression trajectory. **d-f**, Disrupted-interaction genes found in unaffected siblings do not present the above features. **g**, Disrupted-interaction genes are most highly expressed in neuronal cell types. The heatmap shows the negative log10(P-value) for comparing expression levels of disrupted versus non-disrupted interaction genes in seven cell types. Cell types that are significant after Bonferroni correction are noted with their negative log10(P-value) written in white. Abbreviations: pcw, post-conception weeks; mos, moths; CTS, cell-type-specific; NPC, neuronal progenitor cell; ExN, excitatory neuron; InN, inhibitory neuron; Astro, astrocyte; Oligo, oligodendrocyte; Endo, endothelial.

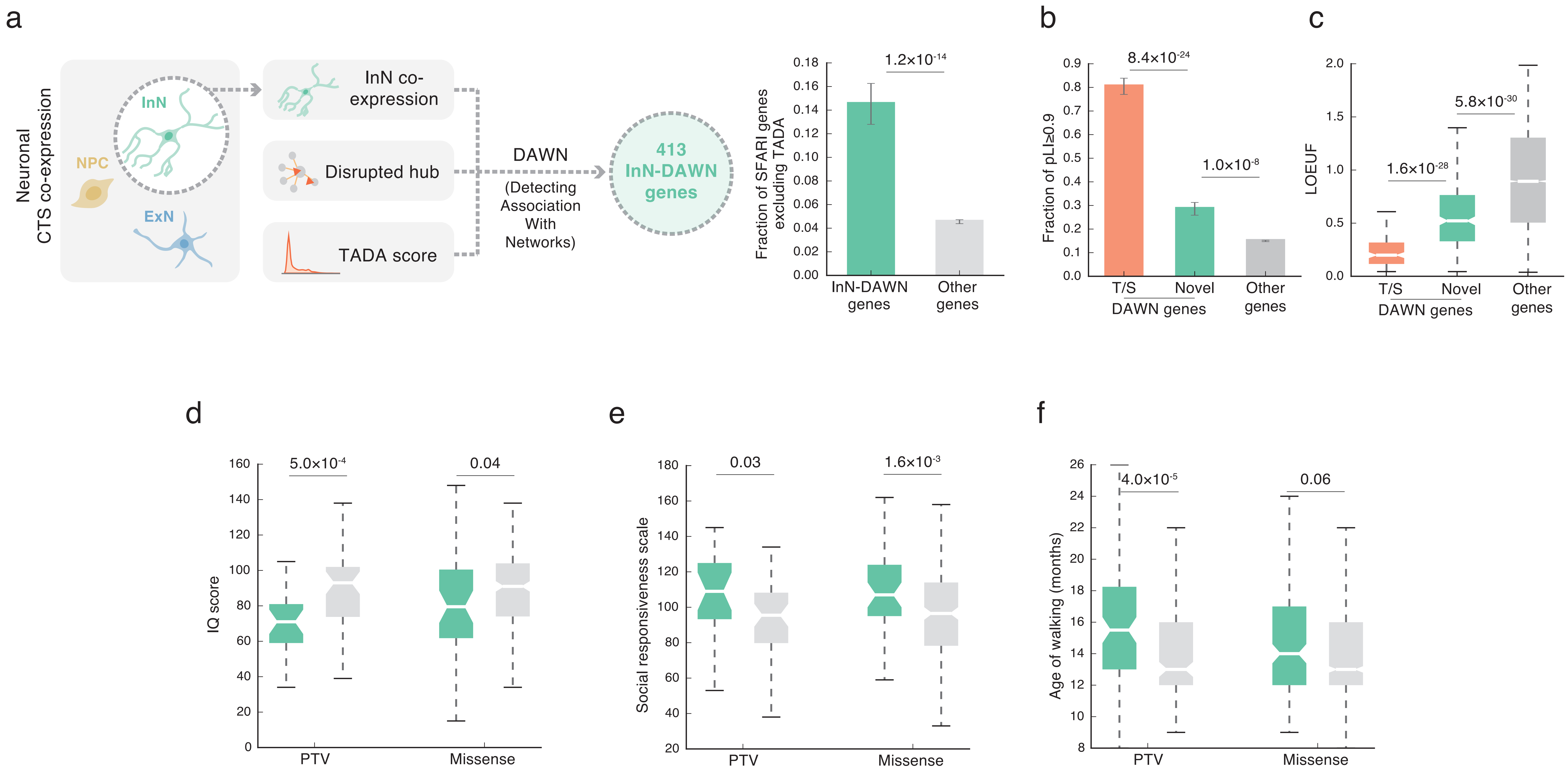

Supplementary Fig. 2: DAWN integrates InN co-expression network to implicate ASD genes in InN. **a**, InN-DAWN genes are enriched for previously implicated ASD genes. **b-c**, InN-DAWN genes versus other genes evaluated on two loss-of-function tolerance metrics, pLI (**b**) and LOEUF (**c**). T/S stands for TADA/SFARI genes also identified by DAWN, Novel for DAWN genes not found in the T/S list, Other genes for expressed genes not found in the T/S or DAWN lists. **d-f**, ASD probands carrying a de novo protein-truncating or missense variant on InN-DAWN genes exhibit severe ASD symptoms, with (**d**) reduced intelligence, (**e**) impaired social ability, and (**f**) delayed age of walking. Abbreviations: CTS, cell-type-specific; ExN, excitatory neurons; InN, inhibitory neurons; NPC, neural progenitor cells; TADA, transmission and de novo association; DAWN, detecting association with networks; pLI, probability of being loss-of-function intolerant; LOEUF: loss-of-function observed/expected upper bound fraction; PTV, protein truncating variant.

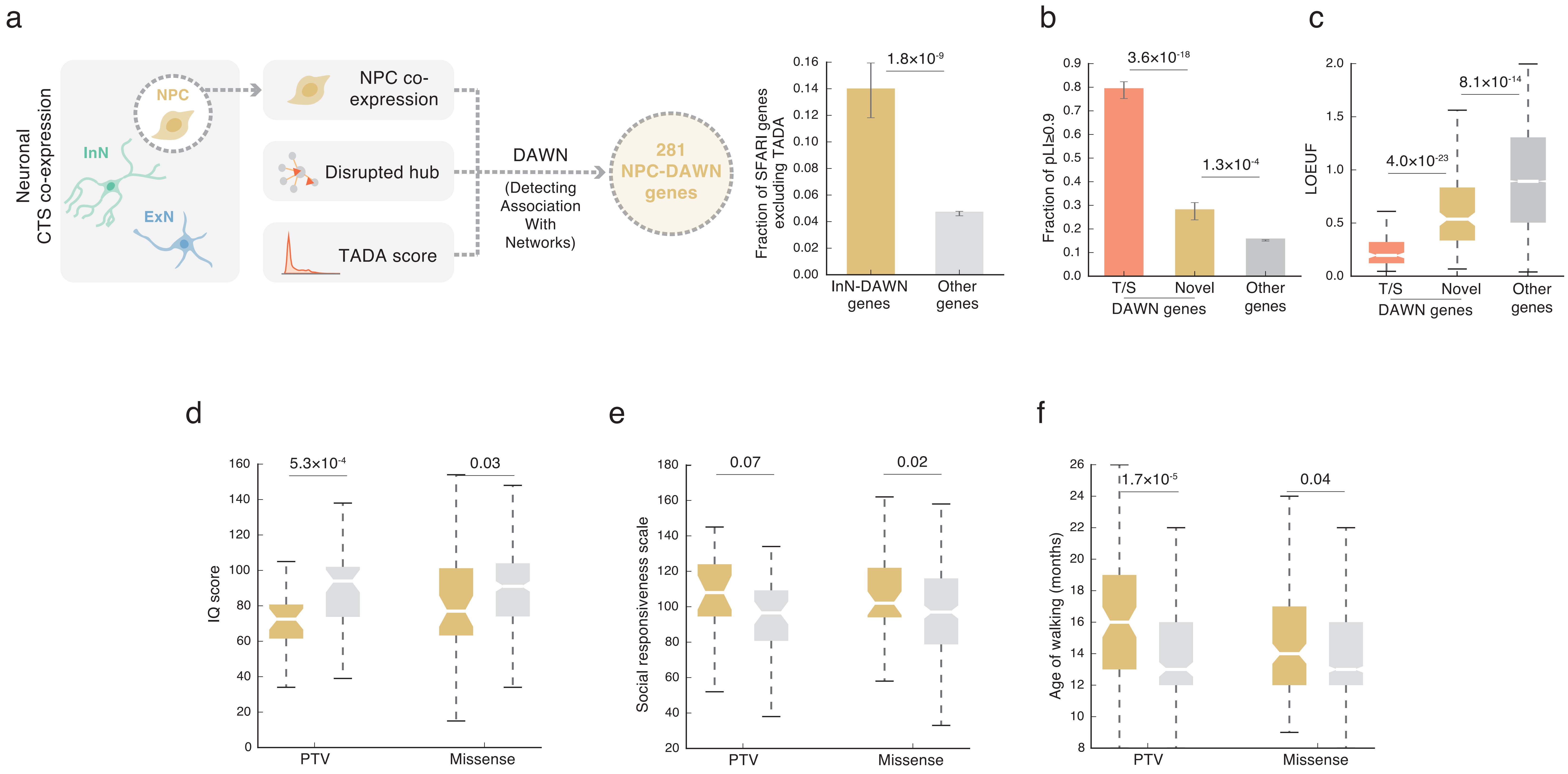

Supplementary Fig. 3: DAWN integrates NPC co-expression network to implicate ASD genes in NPC. **a**, NPC-DAWN genes are enriched for previously implicated ASD genes. **b-c**, NPC-DAWN genes versus other genes evaluated on two loss-of-function tolerance metrics, pLI (**b**) and LOEUF (**c**). T/S stands for TADA/SFARI genes also identified by DAWN, Novel for DAWN genes not found in the T/S list, Other genes for expressed genes not found in the T/S or DAWN lists. **d-f**, ASD probands carrying a de novo protein-truncating or missense variant on NPC-DAWN genes exhibit severe ASD symptoms, with (**d**) reduced intelligence, (**e**) impaired social ability, and (**f**) delayed age of walking. Abbreviations: CTS, cell-type-specific; ExN, excitatory neurons; InN, inhibitory neurons; NPC, neural progenitor cells; TADA, transmission and de novo association; DAWN, detecting association with networks; pLI, probability of being loss-of-function intolerant; LOEUF: loss-of-function observed/expected upper bound fraction; PTV, protein truncating variant.
